# Pembrolizumab-Induced Adrenal Insufficiency Presenting Eight Months After Cessation of Treatment

**DOI:** 10.7759/cureus.41049

**Published:** 2023-06-27

**Authors:** Stephanie Zilberman, Daniel C Rafii, Judith Giunta

**Affiliations:** 1 Internal Medicine, New York-Presbyterian Brooklyn Methodist Hospital, Brooklyn, USA; 2 Endocrinology, Diabetes, and Metabolism, New York-Presbyterian Brooklyn Methodist Hospital, Brooklyn, USA

**Keywords:** prostate cancer, low cortisol, central adrenal insufficiency, cortisol, adrenal insufficiency, pembrolizumab side effect, pembrolizumab

## Abstract

Pembrolizumab is a monoclonal antibody that functions as an immune checkpoint inhibitor. It is an FDA-approved immunotherapy used to treat various malignancies. With its wide use in cancer therapy, there are many known side effects. It is a common cause of endocrinopathies such as thyroid disease and adrenal insufficiency (AI). AI has been most commonly reported during active treatment cycles with pembrolizumab, as well as quickly after the termination of treatment. However, we describe a case of pembrolizumab-induced AI with an onset at eight months following the discontinuation of treatment and discuss prompt treatment when AI is diagnosed.

## Introduction

Pembrolizumab is a monoclonal antibody that blocks the programmed death receptor-1 (PD-1)/programmed death ligand 1 (PD-L1) pathway. It is a common immunotherapy successfully used to treat various malignancies including non-small cell lung cancer, colorectal cancer, bladder cancer, endometrial cancer, and prostate cancer. With its widespread use, we have seen an increase in reported cases of endocrinopathies, including adrenal insufficiency (AI) [1]. AI most commonly presents during acute treatment with pembrolizumab, and much less commonly after cessation of treatment. Here, we present a case of acute AI with an onset at eight months following the cessation of treatment with pembrolizumab. 

## Case presentation

We present a 66-year-old male with a past medical history of type 2 diabetes mellitus, hypothyroidism, and *BRCA*-positive metastatic castration-resistant prostate cancer, which was initially diagnosed three years prior to presentation. He received treatment with pembrolizumab, along with hormonal therapy, for approximately 20 months, which was then stopped due to stable disease. The patient presented to the emergency department eight months following the cessation of pembrolizumab due to an episode of syncope that was associated with a month-long history of decreased oral intake, nausea, vomiting, abdominal pain, and extreme fatigue. He denied fever, headache, chest pain, or shortness of breath. At the time of presentation, the patient’s medications included metformin, Jardiance, and levothyroxine. His family history was positive for heart disease in his brother and he denied family history of autoimmune disease. His vital signs were within normal limits and he was afebrile. Physical exam was significant for a lethargic obese male with dry oral mucosa and abdominal tenderness. Laboratory values were notable for a sodium level of 137 mmol/L and an elevated potassium level of 5.9 mmol/L. A viral respiratory panel was negative for acute infection, including coronavirus disease 2019 (COVID-19), and he tested negative for HIV. Electrocardiogram showed normal sinus rhythm with a prolonged QTC of 652 milliseconds, which was attributed to his ondansetron use and thus subsequently discontinued with improvement in QTC.

The following morning, cortisol and adrenocorticotropic hormone (ACTH) levels were obtained (Table 1). Cortisol and ACTH levels both resulted low; early morning cortisol was 0.34 ug/dL (normal range 6.02-18.4 ug/dL) and ACTH was 3.9 pg/ml (normal range 7.2-63.3 pg/ml). The thyroid stimulating hormone (TSH) level was elevated at 21.6 mIU/L (normal range 0.36-3.74 mIU/L) and free thyroxine was low at 0.2 ng/dL (normal range 0.9-1.7 ng/dL). To note, the patient had not been taking levothyroxine for several weeks due to emesis. Additionally, luteinizing hormone (LH), follicle-stimulating hormone, insulin-like growth factor 1 (IGF-1), prolactin, and growth hormone (GH) levels were obtained and were all within normal limits. He underwent a cosyntropin stimulation test with intravenous cosyntropin 250 micrograms followed by 30- and 60-minute cortisol levels drawn. Both cortisol levels were low at 5.52 ug/dL and 6.93 ug/dL, respectively, leading to a diagnosis of secondary AI due to prior pembrolizumab use, as other causes were ruled out.

**Table 1:** Laboratory values and references ranges ACTH: adrenocorticotropic hormone; TSH: thyroid stimulating hormone

	Lab Value	Normal Range
Early morning cortisol	0.34 ug/dL	6.02 - 18.4 ug/dL
ACTH	3.9 pg/ml	7.2 - 63.3 pg/ml
TSH	21.6 mIU/L	0.36 - 3.74 mIU/L
Free thyroxine	0.2 ng/dL	0.9 - 1.7 ng/dL
Cosyntropin stimulation test, 30-minute cortisol level	5.52 ug/dL	> 18 ug/dL indicates normal adrenal gland response
Cosyntropin stimulation test, 60-minute cortisol level	6.93 ug/dL	> 18 ug/dL indicates normal adrenal gland response

The patient was started on IV methylprednisolone 10 mg every eight hours and after initiation of steroids, his home dose of levothyroxine 125 mcg daily was resumed. The following day, after receiving two doses of glucocorticoids, the patient’s lethargy substantially improved and was without nausea or vomiting. He was discharged home on oral hydrocortisone 25 mg every eight hours. On subsequent endocrinology follow-up, the patient appeared to be back to his baseline activity level. Thus, his oral hydrocortisone dose was tapered to a minimal dose of 10 mg twice a day with continued improvement in symptoms. 

## Discussion

Pembrolizumab is an immune checkpoint inhibitor that binds to the PD-1 receptor on the surface of immune T-cells in order to block PD-L1 from binding [1]. This prevents cancer cells from suppressing the immune system by reversing T-cell suppression and inducing antitumor responses. Pembrolizumab has been approved by the FDA to treat various malignancies as stand-alone and adjuvant therapy, including non-small cell lung cancer, colorectal cancer, bladder cancer, endometrial cancer, melanoma, and prostate cancer with *BRCA* mutations. 

Endocrinopathies are well-documented side effects of pembrolizumab, including primary and secondary AI [2]. Clinical features of acute AI include hypotension, nausea, vomiting, abdominal pain, fatigue, and confusion, with metabolic derangements such as hyponatremia, hyperkalemia, and hypoglycemia [3]. As described by Bornstein et al. [4], and as seen in our patient, AI is suspected when the early morning cortisol level is less than 5 μg/dL, followed by dynamic testing via the cosyntropin stimulation test. An injection of 250 μg IV/intramuscular (IM) of cosyntropin (ACTH) is given, with cortisol levels drawn 30 and 60 minutes post injection; failure to obtain a cortisol level at or above 18 μg/dL is suggestive of AI [4]. 

Pembrolizumab-induced AI is most commonly caused by hypophysitis, which is an inflammation of the pituitary gland, resulting in an ACTH deficiency and subsequent symptoms of AI [5]. This was the likely mechanism in our patient’s case as his ACTH and cortisol levels were both low, and he had improvement in symptoms with the initiation of steroid therapy. An MRI brain was not obtained to visualize hypophysitis; however, brain imaging is not required to confirm the diagnosis. The onset of AI due to the checkpoint inhibitor can occur at any time throughout treatment [6]. The case we present is unique in that the patient presented with AI eight months following the cessation of treatment. Upon literature review, there are very few cases documenting a diagnosis of AI following treatment withdrawal. In a case series by Sonehara et al., five out of 49 patients developed AI, and all five of the patients presented with symptoms while receiving treatment with pembrolizumab [7]. In a retrospective study by Kurokawa et al., nine out of 10 patients were diagnosed with AI while being treated with pembrolizumab and only one patient developed AI after pembrolizumab was discontinued; in their case, it was 4.5 months later [8]. Two additional case reports reported AI diagnosis following treatment withdrawal of pembrolizumab. One case reported acute AI one week after completing eight cycles of immunotherapy, whilst another report demonstrated AI four weeks following the last treatment cycle [9,10]. 

We highlight the importance of recognizing AI if a patient has a history of treatment with pembrolizumab, as it can be an easily missed diagnosis due to the non-specific symptoms. Prompt treatment with oral glucocorticoids is recommended to avoid precipitating adrenal crisis, which can cause severe hypotension and lead to death if not recognized [11]. In patients that experience AI during treatment with pembrolizumab, most are able to continue their treatment cycles if treated with glucocorticoids. In the few cases that reported AI when they were no longer treated with the immune checkpoint inhibitor, they were also treated with glucocorticoids with vast improvement, as seen in our patient. In conclusion, our case revealed an uncommon presentation of diagnosing AI several months after treatment discontinuation. Thus, we propose the idea of post-treatment follow-up with cortisol levels in patients previously on pembrolizumab.

## Conclusions

Pembrolizumab, an immune checkpoint inhibitor, is a widely used immunotherapy for the treatment of various malignancies that continues to have great clinical significance. However, the wide range of endocrinopathy side effects can greatly affect a patient's quality of life and can result in cessation of treatment if it occurs during a treatment cycle. AI is one side effect profile that may potentially be missed by clinicians both during and after treatment with pembrolizumab. We thus present this rare case of delayed onset of AI after discontinuation of pembrolizumab to help aid clinicians be aware of this possible complication and avoid significant morbidity and mortality. 
